# Cloning, Overexpression and *in vitro * Antifungal Activity of *Zea Mays* PR10 Protein

**DOI:** 10.15171/ijb.1357

**Published:** 2017-03

**Authors:** Niloofar Zandvakili, Mohammadreza Zamani, Mostafa Motallebi, Zahra Moghaddassi Jahromi

**Affiliations:** Department of Plant Molecular Biotechnology, Institute of Agricultural Biotechnology (IAB), NIGEB, Tehran, 14965/161, Iran

**Keywords:** Pathogenesis related proteins, PR10, Antifungal activity, Bioassay, *Zea mays*

## Abstract

**Background:**

Plants have various defense mechanisms such as production of antimicrobial peptides, particularly pathogenesis related proteins (PR proteins). PR10 family is an essential member of this group, with antifungal, antibacterial and antiviral activities.

**Objective:**

The goal of this study is to assess the antifungal activity of maize PR10 against some of fungal phytopathogens.

**Materials and Methods:**

*Zea mays PR10* gene (TN-05-147) was cloned from genomic DNA and cDNA and overexpressed in *Escherichia coli*. The existence of a 77- bp intron and two exons in *PR10* was confi rmed by comparing the genomic and cDNA sequences. The *PR10* cDNA was cloned in pET26b (+) expression vector and transformed into *E. coli* strain Rosetta DE3 in order to express PR10 recombinant protein. Expression of the recombinant protein was checked by western analysis. Recombinant PR10 appeared as insoluble inclusion bodies and thus solubilized and refolded. PR10 was isolated using Ni- NTA column. The activity of the refolded protein was confi rmed by DNA degradation test. The antifungal activity of PR10 was assessed using radial diff usion, disc diff usion and spore germination. The hemolytic assay was performed to investigate the biosafety of recombinant PR10.

**Results:**

Recombinant maize PR10 exerted broad spectrum antifungal activity against *Botrytis cinerea, Sclerotinia sclerotiorum, Fusarium oxysporum, Verticillium dahlia* and *Alternaria solani*. Hemolysis biosafety test indicated that the protein is not poisonous to mammalian cells.

**Conclusions:**

Maize PR10 has the potential to be used as the antifungal agent against diff erent fungal phytopathogens. Therefore, this protein can be used in order to produce antifungal agents and fungi resistance transgenic plants.

## 1. Background

Since plants do not have a circulating adaptive immune system, almost always they initiate a complicated network of defense mechanism during pathogen invasion (1). Pathogenesis related proteins (PR) are an example of the best-studied plant defense proteins overexpressed in response to pathogen attacks and systemic acquired resistance (SAR) mechanism (2,3). PR proteins have been recognized and categorized according to their different structures and biological activities. One of the most noteworthy families among these groups is the PR10 family with more than 100 members reported so far. PR10 families are typically acidic proteins of small molecular weight (16-19 kDa) with similar three-dimensional structures, which consist of central b-sheet covered by a-helices on both sides (a-b-a sandwich structure) that cause a compact, bipartite molecular core fixed by hydrophobic interactions and multiple hydrogen bonds. The PRs insensitivity to proteases and their high stability might be due to the compact structure (4-6). Apart from antimicrobial activity, which was detected in the majority of PR families, PR10 proteins are possibly involved in a variety of biological functions including nuclease and some other enzymatic activities in plant secondary metabolisms and plant protection against abiotic stresses (4). In addition, it has been demonstrated that some PR10 proteins control plant growth and development by modulating the endogenous cytokinin level (1,7).

Fungal diseases are counted either as the most important or the second important factor contributing to the yield losses in many substantial crops (8). PR10 family is known as efficient antifungal proteins. It has been reported that ZmPR10 and ZmPR10.1 are two homologues in maize, which are induced by most abiotic stresses such as salicylic acid, CuCl_2_, H_2_O_2_, cold, etiolation, wounding, and biotic stresses such as Aspergillus flavus, Erwinia stewartii and Pseudomonas syringae pv. tomato DC3000 infection (1).

## 2. Objectives

The current study deals with the recombinant expression of *Zea mays* PR10 in *E. coli*, with the goal to investigate its novel antifungal activity against some of the fungal phytopathogens. We report the successful prokaryotic expression, solubilization, refolding, purification and antifungal effects of functionally active PR10 from *Zea mays* (TN-05-147).

## 3. Materials and Methods

### 3.1. Genomic DNA and RNA Isolation

In order to make a comparison between genomic and cDNA of PR10, genomic DNA was extracted from the leaves of *Zea mays* with commercial source (TN-05-147), using CTAB as described by Dan *et al*. (9). According to Xie *et al*., PR10 expression was induced by 300 mM NaCl for 30 min (1). Total RNA was isolated from *Z. mays* (TN-05-147) leaves by RNAX-plus kit (Cinagen, Iran) based on the procedure described by manufacturer. The quality of RNA and genomic DNA samples were assessed by agarose gel electrophoresis.

The first strand of cDNA was synthesized with specific reverse primer and RevertAid™ M-MuLV reverse transcriptase based on the method described by manufacturer (Fermantas, Germany). The RNA was denatured at 70°C, cooled slowly at 22°C for 2 min and incubated at 42°C for 1 h and at 70°C for 5 min.

### 3.2. PCR Amplification

*Z. mays* PR10 cDNA and genomic DNA (GenBank: FJ897503.1) were amplified using two primer pairs (PR10.1-bpF2, PR10.1-XhR for cDNA and PR10.1-XaF, PR10.1-SaR for genomic DNA; Table 1). The amplicons were gel purified via High pure PCR product purification kit (Roche, Germany).

**Table 1:** Specific primer names, sequences and 5**ʹ** cloning sites.

**Primer**	**Sequence**	**5ʹ cloning site**
PR10.1-XaF	GCTCTAGAATGGCCTCCACCAACAGC	*Xba* I
PR10.1-SaR	CGAGCTCTAGTTGTAGGCTTCCGG	*Sac* I
PR10.1-bpF2	GGAAGACAACATGGCCTCCACCAACAG	*Bpi* I
PR10.1-XhR	GCTCGAGGTTGTAGGCTTCCGGGTTG	*Xho* I

Both amplicons, cDNA and genomic DNA for PR10, were cloned inpTZ57 R/T (CinnaGen, Iran) to yield pTZNZ1 and pJNZ1, respectively.

### 3.3. Sequencing and Computer Analysis

*Z. mays* PR10 sequences were retrieved from GenBank and along with the sequences obtained in this study used for multiple sequence alignment. Deduced amino acid sequence from PR10 was received by EditSeq at DNASTAR and used for alignment by CLUSTALW with MegAlign at DNASTAR (Madison, WI, USA).

### 3.4. Expression in E. coli

The PR10 cDNA sequence was cloned into the NcoI and XhoI restricted sites of pET26b (+) expression vector (Novagen, Germany) to create pETNZ1. pETNZ1 was transformed into *E. coli* strain Rosetta DE3.

Transformed bacterial cell was grown at 37°C in in 2× TY medium (16 g.L^-1^ bacto-tryptone, 10 g.L^-1^yeast extract and 5 g.L^-1^ NaCl). The medium contained 50 μg.mL^-1^ kanamycin and 1% (w/v) glucose. At OD_600_ 0.8, cells were washed twice to remove glucose and different amounts of isopropyl-b-D-thiogalactopyranoside (IPTG) were added to induce the expression of the recombinant protein. At the same time other treatments including different incubation time and temperatures were considered for expression optimization. Protein expression was monitored using 12% sodium dodecyl sulfate-polyacrylamide gel electrophoresis (SDS-PAGE) according to Laemmli (10). The amount of overexpressed PR10 in each fraction was quantified with a Bio-Rad GS-800 gel densitometer.

To optimize the efficiency of expression, orthogonal arrays of Taguchi was used. The symbolic identification of these arrays shows the main information on the size of experiment, e.g. M16 has 16 trials. Each column includes a number of conditions depending on the level assigned to each factor. In this study all three columns were allocated with different factors, each of which with four levels (Table 2).

**Table 2:** Variable factors and their levels employed in Taguchi method.

**Factors**	**Level 1**	**Level 2**	**Level 3**	**Level 4**
IPTG (mM)	0.2	0.5	0.7	1
Time (h)	2	4	6	16
Temp (°C)	23	28	33	37

Qualitik-4 software (Bloomfield Hills, MI, USA) for automatic design and analysis of Taguchi experiment was used to determine the optimum recombinant protein expression conditions.

### 3.5. Western Bloting

For immuno-detection of the expressed PR10, total protein extraction was electrophoresed on SDS-PAGE, followed by electrotransfer to PVDF (polyvinylidene fluoride) membrane. The immunoblots were developed with antibody against His-tag, based on the manufacturer’s instruction (Roche, USA). The anti-His tag antibody has been conjugated to horseradish peroxidase (HRP). 4-Chloro-1-naphthol was used as a substrate for HRP results in a colored precipitate.

### 3.6. Protein Extraction and Purification

For total protein extraction, after two freeze-thaw cycles, cells were resuspended in 1 ml lysis buffer for each 10 ml cell culture (50 mM NaH_2_PO_4_, 300 mM NaCl, 1 mM PMSF, pH 8) and homogenized by sonifier using 3 mm diameter probe with 210 um amplitude capacity and 70% vibration amplitude. The sonication procedure was performed in 6 cycles for 30 sec with 45 sec intervals. The mixture was centrifuged at 13,000 ×*g* for 30 min at 4°C. The pellet containing the inclusion bodies was washed in three volumes of PBS and centrifuged as above. All steps were performed at 4°C. Based on modified HaukeLilie, *et al*. (11), inclusion bodies, containing the overexpressed PR10, were recovered and resuspended in 100 mM NaH_2_PO_4_, 10 mM Tris-HCl (pH 8.0) containing 8 M urea and incubated at 22°C for 1 h. Urea-soluble proteins were separated from the urea-insoluble fraction by centrifugation (13,000 ×*g*, 30 min) and consumed to refold into the active form. The purity of the protein was assessed by SDS-PAGE.

Renaturation of the protein was performed by dilution in the equal amount of renaturation buffer in 4°C (100 mMTris pH 8.5, 100 mM NaCl, 100 mM glycine, 2.5% (v/v) glycerol and 140 mM mercaptoethanol). The urea was completely removed by gradual dialysis against buffer containing 100 mM Tris pH 8.5 and 100 mM NaCl.

For recombinant protein purification, the renatured protein containing PR10 with 6×-His-tag at its C-terminus was loaded on the Ni–NTA affinity column according to manufacturer’s instruction. The recombinant PR10 concentration was estimated by Bradford method (12).

### 3.7. DNA Degradation

To evaluate the PR10 DNase activity of the refolded recombinant protein DNA degradation assays was carried out according to Xie *et al*. 2010 using 40 mg of the purified recombinant proteins of PR10 as the sample and the protein free buffer as the negative control (1).

### 3.8. Antifungal Assay

For detection of PR10 antifungal activity, 3 different fungal growth inhibitory assays, i.e., radial diffusion, disk diffusion, and spore germination, were used. The obtained results were analyzed with t-test. The tested concentrations of the recombinant protein were 20, 30 and 40 mg, respectively. Elution buffer was consumed as the negative control.

In radial diffusion assay, the area of growth inhibition for antifungal activity based on modified method of Broglie *et al*. (13) was checked using 100 × 15 mm petri plates containing 25 mL of potato dextrose agar (PDA). After the mycelia colony had expanded, 5 mm holes were made at a distance of 2-5 mm away from the rim of the mycelial colony. Different concentrations of purified PR10 protein were added. The plates were incubated at 28°C until mycelia growth has enveloped peripheral hole containing the negative control (protein free buffer) and had produced crescents of inhibition around the holes containing PR10 protein. The fungal species were *B. cinerea, S. sclerotiorum, F. oxysporum, V. dahliaand, A. solani*.

In disc diffusion assay, the effect of PR10 against *B. cinerea, F. oxysporum, V. dahlia and A. solani*, was investigated. The assay was carried out according to the modified method of Nweze *et al*. (14). Sterilized paper discs were placed on the PDA plate. An aliquot of the mixture of purified PR10 with different concentrations and 2 × 10^4^cells.mL^-1^ spore suspension of *B. cinerea, F. oxysporum, V. dahlia and A. Solani* were added to each disc. Plates were incubated at 28°C until spore germination and mycelia growth in negative control discs were observed. Protein free buffer was used as the negative control.

In spore germination assay, the germination inhibition effect of PR10 against *B. cinerea, S. sclerotiorum, F. oxysporum, V. dahlia* and* A. solani* was investigated based on Xu Hu *et al*. 1997 using 15 mg of PR10 as the sample and protein free buffer as the negative control (15).

### 2.9 Hemolytic Assay

Since there are several reports of antimicrobial peptides showing cytotoxic activity against eukaryotic cells, recombinant PR10 was also assayed for hemolytic activity against human erythrocytes. Hemolytic activity of PR10 was assessed based on Park *et al*. (16). Human red blood cells in PBS (A_blank_) and in 0.1% (v/v) Triton X-100 (A_triton_) were used for the negative and positive controls, respectively. Also, the hemolytic activity of protein free buffer was measured and compared with PR10 hemolytic activity. The obtained results were analyzed with t-test. The percent hemolysis was calculated according to the equation:

Hemolysis% = [(A _sampel_-A _blank_)/(A_triton_-A_blank_)] ×100

## 4. Results

### 4.1 PR10cDNA Cloning and Sequence Analysis

The 498 bp and 574 bp PCR products were amplified from cDNA and genomic DNA of *Z. mays* (TN-05-147) leaf using specific primers as stated above. The amplicons were cloned in cloning vector.

**Figure 1:**
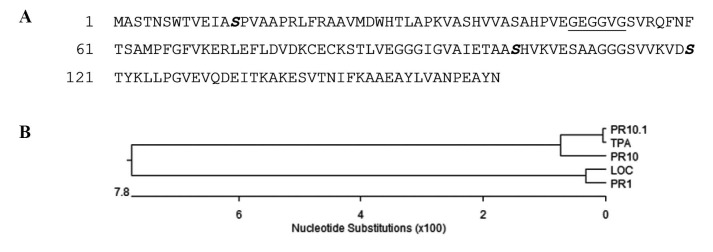
Amino acid sequence of *PR10* cDNA. The P-loop motif is underlined and the putative phosphorylation sites are marked in bold italics (A). Phylogenetic tree obtained using CLUSTALW by MegAlign at DNASTAR for comparing the PR10 amino acid sequence with TPA (DAA44938.1), ZmPR10.1 (ADA68331.1), LOC100192117 (NP_001131012.1) and PR1 (ACG29538.1) (B).

Comparison between the cloned cDNA and genomic DNA indicated that PR10 contains one 77-bp intron and two exons of 483 bp in total, encoding a peptide of 160 amino acids with ~16854.13 Da. A typical GXGGXG motif was evident at amino acid residues 48-53 (Fig. 1A, underlined) of PR10, known as the ‘‘P-loop’’ (phosphate-binding loop), which was reported to be frequently seen in protein kinases and nucleotide-binding proteins (17).

Multiple sequence alignment of the deduced amino acid with related proteins, TPA (DAA44938.1), ZmPR10.1 (ADA68331.1), LOC100192117 and PR1 (ACG29538.1) was performed using CLUSTALW. Alignment showed very high sequence homology (more than 87%; Fig. 1B). Signal peptide was absent in all proteins.

### 4.2 Prokaryotic Expression

The cDNA of PR10 was isolated from pTNZ1 by enzymatic digestion utilizing *Bpi* I and *Xho* I, and sub cloned in pET26 b(+) prokaryotic expression vectorwith an inbuilt His_6_-tag.The recombinant protein was overexpressed in *E. coli* Rosetta (DE3), which supplies tRNA genes for rare codons. In order to optimize the recombinant protein expression, M16 orthogonal experimental design was used to examine the effect of induction time and temperature, and IPTG concentration. The experiments were managed using four levels for each factor.

**Figure 2:**
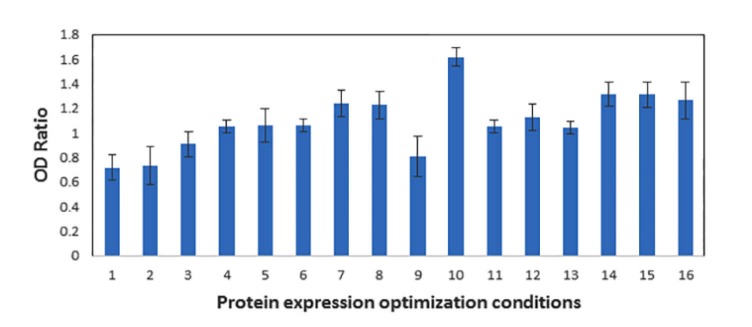
Quantitative analysis of protein expression optimization conditions. 1-16: Diff erent trials used in M16 orthogonal experimental design.

The 17.9 kDa protein band was observed on SDS-PAGE and the amount of expressed protein was estimated via densitometry using Qualitek-4 software (Fig. 2). The influence of each factor on the recombinant protein expression was shown in Table 3A. When the interactions of different factors were calculated (Table 3B), induction time and temperature exhibited the highest interaction severity index (SI) with 48.39%. The optimum conditions for protein expression were 16 h of induction with 1 mM IPTG at 37°C.

**Table 3:** Qualitek-4 analysis: A) Main factors (average effects of factor and interactions).

** A) **
Factor	Level 1	Level 2	Level 3	Level 4
IPTG (mM)	-1.69	1.075	0.84	1.712
Time (h)	-1.139	0.954	0.884	1.238
Temp (°C)	-0.071	0.106	0.225	1.677
** B) **
Interaction	Factor Pairs	Columns*	SI (%) ** Col.	Opt.***
Temp × Time	2 × 3	48.39	1	[2, 4]
IPTG × Temp.	1 × 2	1.22	3	[3, 2]
IPTG × Time	1 × 3	0.73	2	[3, 4]

* Columns – Indicate the column locations to which the interacting factors are determined. ** SI – Interaction severity index (100% for 90 degrees angle between the lines, 0% for parallel lines). *** Opt – Shows factor levels suitable for the optimum condition.

**Figure 3:**
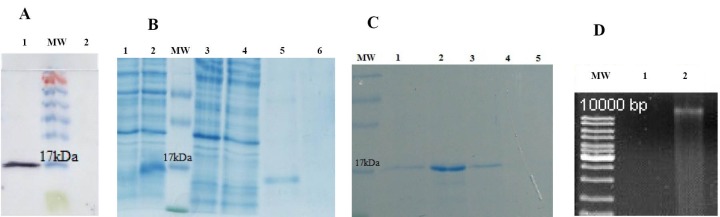
(A) Western blotting. 1: Recombinant PR10. 2: Negative control (extract protein from *E. coli* cells transformed with empty vector). (B) Protein localization. 1, 4 and 6: Negative controls (extract protein from *E. coli* cells transformed with empty vector). 2, 3 and 5: Protein expression of *E. coli* carrying pETNZ1. 1, 2: Total protein. 3, 4: soluble proteins. 5, 6: insoluble proteins (inclusion bodies). (C) purified recombinant PR10. 1: MES, 2, 3: elution, 4: wash, 5: fl ow through. (D) DNA degradation test. 1:DNA treated with recombinant PR10. 2: Negative control (protein free buff er). A, B, C and D MW: molecular marker (Thermo Scientifi c, SM0671, Germany)

The expression of protein was confirmed by western blot (Fig. 3A). The majority of recombinant PR10 was not soluble in water or low salt buffers and expressed as inclusion body (Fig. 3B). *E. coli* cells transformed with an empty vector was considered as negative control (Fig. 3A, B).

The inclusion bodies were denatured in the presence of 8 M urea and refolded by removing urea gradually through dialysis. The refolded recombinant PR10 was purified using Ni-NTA affinity chromatography column (Fig. 3C). The activity of purified refolded PR10 was confirmed by its clear DNase activity against the maize genomic DNA. The protein free buffer was used as negative control (Fig. 3D).

### 4.3 Antifungal and Hemolytic Assays

**Figure 4:**
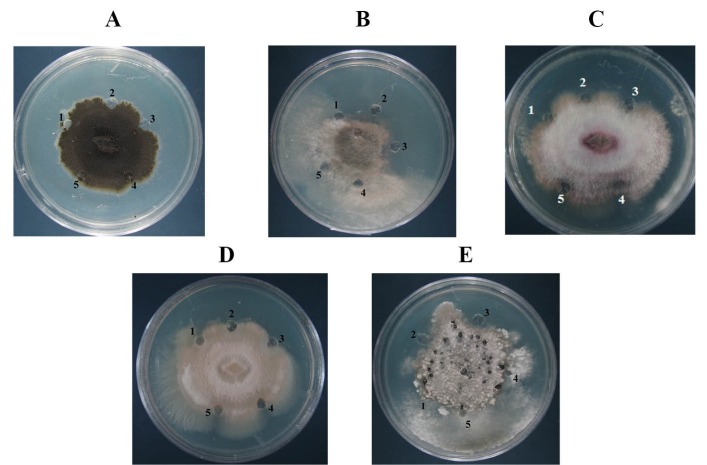
Radial diff usion assay. (A) *A. solani*, (B) *B. cinerea*, (C) *V. dahlia*, (D) *F. oxysporum*, (E) *S. sclerotiorum *. 1-3: Treated with 20, 30 and 40 μg of recombinant PR10, 4 and 5: PR10 free buff er as the negative control

**Figure 5:**
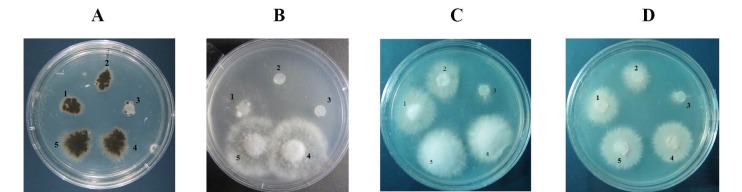
Disc diff usion assay. (A) *A. solani*, (B) *B. cinerea*, (C) *V. dahlia*, (D): *F. oxysporum*. 1-3: Treated with 20, 30 and 40 μg of recombinant PR10.

The antifungal activity of the refolded purified PR10 was investigated using numbers of assays. The purified PR10 protein showed an inhibitory effect on conidia germination and hyphal growth of *B. cinerea, S. sclerotiorum, F. oxysporum, V. dahlia* and* A. solani.* The conidia germination and hyphal growth in radial and disc diffusion assays were decreased by increasing the concentration of purified PR10 (Figs. 4 and 5). Furthermore, the fungi tested in spore germination assay appeared to be sensitive to 15 mg of PR10.

These results demonstrated the inhibition effect of recombinant PR10 on the growth of tested fungi in a concentration dependent manner.

No significant hemolytic activity (<0.5%) at a concentration of up to 60 mg of recombinant PR10 was observed.

## 5. Discussion

PR10 family is an important member of pathogenesis related proteins, which has a significant role in plant defense mechanism against a variety of biotic and abiotic stress. For instance, some of tobacco PRs were recognized as chitinases and b-1,3-glucanases with potential of antifungal activity (18-20). Thus, we have examined *in vitro* antifungal activity of recombinant PR10.

PR10 cDNA and gene were isolated from *Z. mays* (TN-05-147) leaf. The comparison between cDNA and genomic DNA sequences of PR10, showed the existence of 77 bp intron, which is similar to ZmPR10.1 (1). The comparison of deduced amino acid sequence of PR10 and the related amino acid sequences indicated a high degree of homology. According to phylogenic tree (Fig. 1-B), the ancestor of PR10 and ZmPR10.1 and TPA was the same. Furthermore, the same residues for potential phosphorylation sites in all related amino acids were evident demonstrating a probable structural and functional similarity (1,21). In addition, similar to the other members of PR10 family, the lack of signal peptide indicated their intracellular localization (1).

It is perceived that recombinant proteins overexpression in bacteria usually lead to form insoluble proteins containing most of the expressed protein (3). Although the expressed ZmPR10.1 was soluble, ZmPR10 and the recombinant PR10 obtained in this study were formed into insoluble inclusion bodies (1,21). It is assumed that the lack of post translational modification in bacteria (phosphorylation) may cause such result (22). Recombinant PR10 solubilized inclusion bodies were successfully renatured by slow dialysis that followed by affinity column purification that resulted in a functionally active PR10. The activity of refolded purified PR10 was confirmed by the maize genomic DNA degradation. The same result about the effect of ZmPR10.1 on the maize genomic DNA was reported by Xie *et al*. (1).

Both recombinants, ZmPR10 and ZmPR10.1, exhibited antifungal activity against A. flavus (1,21). In addition, ZmPR10 inhibits the growth of V. dahlia (21).

The comparison of conidia germination and fungal growth in the presence of various concentrations of recombinant purified PR10 revealed that it has the potential to inhibit conidial germination and hyphal growth of A. solani, B. cinerea, V. dahlia, F. oxysporum, S. sclerotiorum. Although the recombinant PR10 concentration for fungal growth inhibition was varied, 40 mg of this protein was enough to make inhibitory zone for all tested fungi. The antifungal effect of PR10 proteins is probably due to inhibition of hyphal growth, spore lysis and/or reduction in spore germination or viability of germinated spores. However, the mechanism by which PR10 proteins bring about these effects has not been completely understood (1,7,21)(1,16).

Considering antifungal activity of *Z. mays* PR10 against broad spectrum of phytopathogenic fungi, this protein can be used to develop fungal resistant crop plants.

So the results presented here will be useful to achieve this aim. So hemo-compatibility of PR10 protein should be considered since it might be applied by human in antifungal drugs or transgenic plants. The hemo-compatibility of PR10 was demonstrated as it showed no hemolytic activity.

## Acknowledgments

This research was supported by National Institute of Genetic Engineering and Biotechnology (NIGEB).
